# Experimental evolution supports signatures of sexual selection in genomic divergence

**DOI:** 10.1002/evl3.220

**Published:** 2021-03-22

**Authors:** R. Axel W. Wiberg, Paris Veltsos, Rhonda R. Snook, Michael G. Ritchie

**Affiliations:** ^1^ Centre for Biological Diversity University of St Andrews St Andrews KY16 9TH United Kingdom; ^2^ Current Address: Department of Environmental Sciences Zoological Institute University of Basel Basel CH‐4051 Switzerland; ^3^ Department of Ecology and Evolutionary Biology University of Kansas Lawrence Kansas 66045; ^4^ Department of Zoology Stockholm University Stockholm 106 91 Sweden

**Keywords:** *Drosophila pseudoobscura*, experimental evolution, *F*_ST_, genomic divergence, genomic islands, sexual selection, Tajima's *D*, X chromosome divergence

## Abstract

Comparative genomics has contributed to the growing evidence that sexual selection is an important component of evolutionary divergence and speciation. Divergence by sexual selection is implicated in faster rates of divergence of the X chromosome and of genes thought to underlie sexually selected traits, including genes that are sex biased in expression. However, accurately inferring the relative importance of complex and interacting forms of natural selection, demography, and neutral processes that occurred in the evolutionary past is challenging. Experimental evolution provides an opportunity to apply controlled treatments for multiple generations and examine the consequent genomic divergence. Here, we altered sexual selection intensity, elevating sexual selection in polyandrous lines and eliminating it in monogamous lines, and examined patterns of allele frequency divergence in the genome of *Drosophila pseudoobscura* after more than 160 generations of experimental evolution. Divergence is not uniform across the genome but concentrated in “islands,” many of which contain candidate genes implicated in mating behaviors and other sexually selected phenotypes. These are more often seen on the X chromosome, which also shows greater divergence in *F*_ST_ than neutral expectations. There are characteristic signatures of selection seen in these regions, with lower diversity on the X chromosome than the autosomes, and differences in diversity on the autosomes between selection regimes. Reduced Tajima's *D* within some of the divergent regions may imply that selective sweeps have occurred, despite considerable recombination. These changes are associated with both differential gene expression between the lines and sex‐biased gene expression within the lines. Our results are very similar to those thought to implicate sexual selection in divergence between species and natural populations, and hence provide experimental support for the likely role of sexual selection in driving such types of genetic divergence, but also illustrate how variable outcomes can be for different genomic regions.

**Impact summary**

How does sexual selection contribute to the divergence of genomes? It is often thought that sexual selection is a potent force in evolutionary divergence, but finding “signatures” of sexual selection in the genome is not straightforward, and has been quite controversial. Here, we used experimental evolution to allow replicate populations of fruit flies to evolve under relaxed or strengthened sexual selection for over 160 generations, then sequenced their genomes to see how they had diverged. The features we find are very similar to those reported in populations of natural species thought to be under strong sexual selection. We found that genomic divergence was concentrated in small patches of the genome rather than widespread. These are more often seen on the X chromosome, which overall shows greater divergence than autosomes. There is also lower genetic diversity in these regions, which is a characteristic signature of strong selection. The changes are associated with both differential gene expression between the lines and sex‐biased gene expression within the lines. Many of the patches of divergence also contain candidate genes implicated in mating behaviors and other sexually selected phenotypes. Our results provide experimental support for the likely role of sexual selection in driving such types of genetic divergence.

The role of sexual selection in influencing evolutionary divergence and speciation is unclear (Panhuis et al. 2001; Ritchie 2007; Maan and Seehausen 2011; Servedio and Boughman 2017). Associations between species diversity and proxies of sexual selection such as sexual dimorphism or mating system variation often imply that sexual selection can accelerate divergence, especially when acting alongside natural selection (Arnqvist et al. 2000; Gage et al. 2002; Ellis and Oakley 2016). However, different indicators of sexual selection give contrasting results in such comparative studies, and a consensus is not clear (Kraaijeveld et al. 2011; Janicke et al. 2018). One potentially compelling source of evidence that sexual selection is involved in divergence is coming from the increasing number of comparative genomic studies available across a range of organisms. Many descriptions of genomes, including those of species thought to have undergone strong sexual selection such as the Hawaiian *Drosophila* or African cichlids, have found that genes associated with mating behavior or sensory perception potentially involved in sexual communication are often outliers in measures of divergence (e.g., Mattersdorfer et al. 2012; Kang et al. 2016). It has also been known for some time that genes that diverge particularly rapidly and show stronger signatures of positive divergent selection are often sex biased in expression (Pröschel et al. 2006; Ellegren and Parsch 2007; Zhang et al. 2007). Sex‐biased gene expression itself, especially male‐biased expression, evolves rapidly and this is associated with indicators of sexual selection such as increased sexual dimorphism in birds (Harrison et al. 2015; Wright et al. 2019). However, genes with sex‐biased gene expression might experience more drift than unbiased genes, either due to reduced pleiotropy (Gershoni and Pietrokovski 2014; Allen et al. 2018) or because they experience only half the selection pressure of genes with unbiased expression (Dapper and Wade 2020). Additionally, divergence of sex chromosomes between species is usually much greater than autosomes, sometimes dramatically so (Counterman et al. 2004; Ellegren et al. 2012).

Such patterns of divergence are not necessarily driven by elevated sexual selection on these genes or genomic regions. Sex‐biased gene expression is thought to evolve due to sexually antagonistic selection on gene expression, which is an important factor in sexual selection but can arise due to other types of sex‐specific selection. Changes in sex bias in gene expression are also complicated by additional factors including dosage compensation, turnover of sex‐biased expression, and resolution of conflict via sex‐linkage or sex‐limited expression (Mank et al. 2010a; Wright et al. 2019). The increased divergence of sex chromosomes is also potentially influenced by many factors, including a greater role of genetic drift due to a smaller effective population size of X chromosomes compared to autosomes, dominance effects, and other consequences of sex linkage such as dosage compensation (Vicoso and Charlesworth 2006; Ellegren 2009; Mank et al. 2010b). Hemizygosity results in a lower effective population size (*N*_e_) on the X (*N*_eX_) than on autosomes (*N*_eA_). Under random mating, the ratio of *N*_e_ is expected to be 3:4 and this should reduce neutral diversity and increase between‐species divergence by the same proportion (Vicoso and Charlesworth 2006). Hemizygosity should also result in an increased efficacy of selection for partially recessive beneficial mutations on the X‐chromosome, relative to autosomes, and against recessive deleterious mutations on the X, relative to autosomes. Finally, because of the female‐biased inheritance patterns of X‐linked loci (males transmit them only to daughters, whereas females transmit them to both daughters and sons), sex‐limited selection as well as sexual selection will influence their divergence (Mank et al. 2010a; Corl and Ellegren 2012; Wright et al. 2015).

It is very difficult to infer the historical role of different evolutionary processes from patterns of contemporary divergence between populations and species, because they can result in similar genomic signals (Butlin et al. 2012). One way of directly addressing the role of sexual selection or mating system variation in genomic divergence is to examine the genomic consequences of experimental evolution under manipulated sexual selection regimes in the laboratory. A great advantage of this approach is that there are potentially fewer confounding variables involved than when making comparisons across species or natural populations. However, a disadvantage is that the time scale over which divergence can be studied is typically much shorter than evolutionary timescales in nature. Studies of experimental evolution and speciation are in their infancy, most have studied phenotypic rather than genomic divergence, and general conclusions are, as yet, difficult to draw (White et al. 2020). Enforcing monogamy in otherwise polyandrous species will lead to both changes in the intensity of sexual selection and the balance of sexual conflict, as it effectively eliminates sexual selection and sexually antagonistic selection. A classic example of such manipulation is where *D. melanogaster* were kept under enforced monogamy for about 50 generations (Holland and Rice 1999). Females from the monogamy treatment had reduced longevity compared to ancestral females, when exposed to ancestral males. This was expected because the reduction of conflict should favor less harmful males and females that are less resistant to male harm. Other experimental evolution studies under altered mating systems have been performed in dung flies (Hosken et al. 2001; Hosken and Ward 2001; Martin and Hosken 2003), different species of fruit flies (*D. melanogaster* [Gerrard et al. 2013; Hollis et al. 2014; Innocenti et al. 2014; Perry et al. 2016]; *D. pseudoobscura* [Crudgington et al. 2005]; *D. serrata* [Chenoweth et al. 2015]), seed beetles (McNamara et al. 2020), and hermaphroditic flatworms (Janicke et al. 2016). Although aspects of the treatments differ among such experiments, some common patterns have emerged. Gene expression changes are seen, especially of genes that are initially sex biased, although the details can vary between studies (Hollis et al. 2014; Veltsos et al. 2017). Moreover, gene expression changes can be more pronounced for genes expressed in reproductive tissues (Innocenti et al. 2014), and genes involved in the post‐mating physiological manipulation of female egg‐laying and re‐mating rates (Perry et al. 2016).

A feature emerging from genomic comparisons between diverging species is that details of genomic architecture, in particular how genome structure influences variation in recombination rate, complicate the assessment of patterns of divergence across chromosomes. Whole chromosomal regions can show correlated responses due to reduced recombination and hitchhiking effects, especially in species with segregating inversions. Early studies of species differences interpreted “islands” of divergence in the genome as resulting from divergent selection on genes within these regions with gene flow homogenizing the genetic background (Turner et al. 2005; Nosil et al. 2009). More recently, it has been appreciated that chromosomal inversions and other regions of low recombination or diversity can accentuate such clustered divergence (Noor and Bennett 2009; Cruickshank and Hahn 2014; Wolf and Ellegren 2016; Ravinet et al. 2017). “Barrier loci,” genomic regions under divergent selection that restrict gene flow (Butlin and Smadja 2018), may occur within such clusters but the lack of recombination makes them difficult to localize precisely. In experimental evolution, the amount of recombination will be determined by both genomic architecture and the number of generations completed during the study, which is often modest in studies of eukaryotes. Also, in experimental evolution the lines can be kept effectively allopatric, so homogenizing gene flow in regions not experiencing selection should be absent. The genomic divergence that occurs during experimental evolution is usually extensive, with widespread differences dispersed throughout the genome (Kawecki et al. 2012; Tobler et al. 2014; Michalak et al. 2019).

Here, we directly test the influence of sexual selection on genomic (allele frequency) divergence. We examine replicated experimentally evolved lines of *D. pseudoobscura* in which sexual selection has been manipulated for over 160 generations. One set of four replicate lines was raised under enforced monogamy (M lines), which should eliminate both sexual selection and conflict. Another four replicates were reared under elevated polyandry (E lines), with six males per female. Polyandry mediates the strength of both intra‐ and intersexual selection and sexual conflict (Pizzari and Wedell 2013) and elevated polyandry will increase both pre‐ and postcopulatory sexual selection via female choice and sperm competition beyond levels experienced in most natural populations (Snook 2014). Previous studies of these lines have found divergence in some, but not all, of the types of traits predicted to diverge under sexual selection. Sperm morphology and heteromorphism, and testis mass did not diverge, but E males had larger accessory glands and a greater mating capacity (Crudgington et al. 2009), were more competitive in mating encounters (Debelle et al. 2016), and produced more attractive courtship song than M males (Debelle et al. 2017). Coevolutionary changes have occurred in female song preferences (Debelle et al. 2014). Sexually dimorphic cuticular hydrocarbons have also diverged between the lines (Hunt et al. 2012).

Patterns of gene expression have also changed between the lines. E females show an increase in expression of genes normally enriched in ovaries (Immonen et al. 2014). Sex‐biased genes responded more strongly to the sexual selection treatment, but the direction of gene expression changes differed between sexes, tissues, and according to courtship experience (Veltsos et al. 2017). In most cases, the transcriptome was “feminized” under polyandry (i.e., female‐biased genes were upregulated or male‐biased genes downregulated in E lines), in a striking contrast to a similar study with *D. melanogaster* (Hollis et al. 2014). Males changed in patterns of gene expression in the testes and accessory glands, and changes in gene expression in females following mating also diverged, especially in the female reproductive tract (Veltsos et al. 2021).

Here, we examine genomic divergence between these lines using a pool‐sequence approach (Schlötterer et al. 2014) after more than 160 generations of experimental evolution. The relatively long timescale of this study should reduce linkage effects on allele frequency changes. We adopt a statistical approach that identifies alleles that have changed in frequency consistently across the replicates, to help reduce the potentially confounding effects of drift or replicate‐specific selection (Wiberg et al. 2017). We find that divergent SNPs are not distributed randomly across the genome, but occur in distinct, obvious clusters. We examine what genes are involved and find several with mutant phenotypes related to mating and courtship behaviors. We found that the X chromosome has accumulated more divergence than the autosomes and explore if divergence is associated with recombination rate or changes in gene expression between the experimental lines.

## Methods

### EXPERIMENTAL EVOLUTION

A full description of the experimental evolution procedure is available elsewhere (Crudgington et al. 2005). Briefly, a population of *D. pseudoobscura* was established from 50 wild caught females, bred in the laboratory for four years, and then four independent monogamy (M) and elevated polyandry (E) lines were established. M females were housed with a single male and E females with six males, with females typically mating with two or three males. The effective population size was maintained around 120 (Snook et al. 2009) for both treatments to try to minimize confounding effects of drift and treatment. At each generation, offspring were collected and pooled together for each replicate line, and a random sample used to constitute the next generation in the appropriate sex ratio, thus reflecting the differential offspring production across families (Crudgington et al. 2005; Crudgington et al. 2009). Enforced monogamy is expected to eliminate sexual selection and sexual conflict, whereas elevated polyandry increases both pre‐ and postmating sexual selection and sexual conflict beyond levels encountered in most natural populations and in the ancestral population (Crudgington et al. 2005; Bacigalupe et al. 2007; Crudgington et al. 2009).

### SEQUENCING AND MAPPING

Sequencing was carried out after approximately 160 generations of selection (specifically, 164 for replicate 1, 163 for replicate 2, 162 for replicate 3, and generation 160 for replicate 4). Two pools of 40 females (one E and one M) were taken from each replicate line and genomic DNA extracted using a standard Phenol‐Chloroform extraction protocol. Each pool was sequenced across two lanes on an Illumina HiSeq platform at the Centre for Genomic Research (CGR) at the University of Liverpool. Details of coverage are provided in the supplementary material. Reads from each sequenced pool were mapped to the *D. pseudoobscura* reference genome (FlyBase version 3.1, February 2013) using BWA mem (version 0.7.7; Li 2013). Alignments were filtered to remove duplicate reads, reads with a mapping quality <30, and any reads that were not properly paired, using samtools (version 1.3; Li et al. 2009 following Schlotterer et al. 2014). Reads were locally re‐aligned around indels using GATK (version 3.7.0; McKenna et al. 2010; DePristo et al. 2011). The .bam files for each line were then merged using bamtools (Barnett et al. 2011) and the genome‐wide coverage was calculated from these merged files with bedtools (version 2.26; Quinlan and Hall 2010). SNPs were called using a heuristic SNP calling algorithm (PoolSNP; Kapun et al. 2020). Sites were considered only if the total coverage at the site was more than 17 and less than the 95^th^ percentile for each contig or chromosome. An allele was only called if the count for that allele across all pools was >16 and the allele frequency across all pools was >0.001. Nearly 2 million SNPs were called and used in downstream analyses (see Supporting Information).

### GENOMIC ANALYSES

#### Identifying consistent allele frequency differences

Many evolve and resequence studies of *Drosophila* find that a multitude of SNPs have diverged, perhaps tens of thousands (Michalak et al. 2019). The number is inflated upward at least in part due to segregating inversions and other areas of low recombination, and hitchhiking (Barghi and Schlotterer 2019). To focus on the loci most likely to have diverged due to the treatment, we only considered as candidate SNPs those that diverged consistently across all four replicate pairs of lines. We identified these using quasibinomial Generalized Linear Models, which are less prone than other statistical approaches to be influenced by strong divergence in only some replicates (Wiberg et al. 2017). The model structure applied was as follows:

y∼treatment+e,

where *y* is the allele frequency of the major allele (identified as the major allele across all pools) within each sample, *treatment* is the experimental evolution treatment regime of each sample, and *e* is a quasibinomially distributed error term. If any count within a population was 0, +1 was added to all counts. *P*‐values were converted to *q*‐values using the “qvalues” R package (version 2.16.0; Storey and Tibshirani 2003). A threshold of 0.05 was chosen to control the false discovery rate (FDR), thus we define “top SNPs” as those that change consistently across all replicates with *q*‐value < 0.05 and the remainder are referred to as “background” SNPs.

#### Genetic diversity

We calculated genome‐wide genetic diversity statistics (π and Tajima's *D*) for windows of 50 kb (with a 10‐kb overlap) using available python scripts (Kapun et al. 2020). Similarly, we computed a value of Tajima's *D* for each annotated *D. pseudoobscura* gene by taking the mean value across all 50‐kb windows that spanned a gene. Comparisons of parameters between selection regimes and genomic regions were tested using nonparametric Wilcoxon tests.

#### Genetic differentiation

We computed pairwise *F*_ST_ estimates between E and M line pairs for each SNP using the R package “poolfstat” (version 0.0.1; Hivert et al. 2018), averaged in windows of 50 kb (with a 10‐kb overlap between windows). *F*_ST_ is not independent of the gene frequency changes analyzed above, but allows comparison with many studies of species differences. We estimated neutral expectations for *F*_ST_ expected from drift and differences in effective population sizes on X chromosomes (*F*_X_) as in Machado et al. (2016) using the equations of Ramachandran et al. (2004) (eq. 8 therein); *F*_X_ is given by

$$F_{X}=1-\left\{\frac{\left(9\left(z+1\right)\left(1-F_{A}\right)\right)}{\left(8\left(2z+1\right)-\left(1-F_{A}\right)\left(7z-1\right)\right)}\right\},$$

where, *z* is the ratio of the number of breeding males to females and *F*_A_ is the observed *F_ST_* on autosomes. We assumed *z* to be either 1 or 6 to represent extreme possibilities based on the mating system manipulation. For each E‐M pairwise comparison, we calculated mean *F*_ST_ across each chromosome type and converted to *F*_X_. We used a bootstrapping approach to obtain a random distribution of *F*_X_ for each replicate. For each of 1000 bootstrap iterations, we sampled, with replacement, a number of windows equal to the total number across all autosomes from the set of all windows, and then we calculated mean *F*_ST_ across all sampled windows and converted to *F*_X_ using the equation above. Additionally, we computed a value of *F*_ST_ for each annotated *D. pseudoobscura* gene by taking the mean value across all 50‐kb windows that spanned a gene.

#### Linkage disequilibrium

Although haplotype information is not available from pool‐seq data, short range linkage information is available from paired reads. We used linkage disequilibrium (LDx) (Feder et al. 2012) to first compute the *r* ^2^ of SNPs located on the same read pairs. We only used SNPs with a minor allele frequency >0.1, a minimum coverage of 10, a maximum read coverage of 400, and a Phred score >20. Note that the empirical median insert size varied between 332 and 346 bp across samples. We computed the pairwise distances between all pairs of SNPs and then computed mean *r* ^2^ per distance class. We filtered to use only distance classes with a minimum of five SNPs. The precise number of final distance classes varied across chromosomes from 245 (chromosome 3) to 280 (chromosome 2). The range of these distance classes also varied across chromosomes with 11–300 bp (chromosomes 2, 3, XL, and XR) and 4–300 bp (chromosome 4). We estimated the decay of *r* ^2^ as a function of distance by fitting a linear model of *r* ^2^ as a function of the log of the distance between the SNPs. Thus, the slope measures the decay rate of linkage due to recombination (Feder et al. 2012), giving an indication of the distance over which LD is present. In regions of low recombination, one would expect high overall values of *r* ^2^ but a weakly negative slope as LD is maintained over relatively longer regions of the genome. Comparing the slope parameter across different genomic regions gives an indication of differences in the recombination rate (or extent of selective sweeps). This was performed for each chromosome, as well as for different regions on the third chromosome (see below).

### GENE FUNCTIONS AND EXPRESSION VARIATION

To examine the function of genes near candidate SNPs, we conducted enrichment analyses. We used the *D. pseudoobscura* annotation and a dataset of regulatory long noncoding RNAs (lncRNAs; Nyberg and Machado 2016). We identified genes or lncRNAs within a distance of 10‐kb up‐ or downstream of top SNPs with bedtools (Quinlan and Hall 2010) intersect (keeping any potential ties). Enhancer regions, transcription factor binding sites, and other regulatory regions can occur up to 1‐Mb up‐ or downstream from a target gene in other species (e.g., Maston et al. 2006; Chan et al. 2010; Werner et al. 2010; Pennacchio et al. 2013) but typically lie within 2 kb of a gene region in *D. melanogaster* (Arnosti 2003); 10 kb thus represents a compromise. We submitted the implicated genes to ModPhEA (Weng and Liao 2017) for phenotypic enrichment analysis. We combined the phenotypic classes “courtship behavior defective” (FBcv:0000399) and “mating rhythm defective” (FBcv:0000401) into one phenotype group and also tested the phenotypic class “stress response defective” (FBcv:0000408) for enrichment. We chose these classes a priori because they were most likely to be involved in phenotypic differences between the treatments related to mating or courtship behavior and responses.

We also took advantage of gene expression data from the same experimental evolution lines. Expression data are available from heads and abdomens of virgin and courted flies (Veltsos et al. 2017) and testes, accessory glands, ovaries and female reproductive tracts from virgin flies, and ovaries and female reproductive tracts from mated females (Veltsos et al. 2021). Using these data, we compiled a list of genes with differential expression between E and M lines. For simplicity, we considered a gene to be differentially expressed between E and M lines if it shows significant differences in E/M contrasts in any of the following data: combined virgin and courted head or abdomens of each sex (four sets), virgin individual reproductive tissues (four sets), and mated individual female reproductive tissues (two sets). Briefly, the analysis was conducted in edgeR version 3.18.1 (Robinson et al. 2010) running in R version 3.4.0 (R Development Core Team 2020). We used TMM normalization in edgeR and measured dispersion using a negative binomial model from the genes within each contrast. We employed a statistical definition for differential expression (FDR < 0.05; Benjamini and Hochberg 1995) and did not require a minimum log_2_FC threshold to consider a gene differentially expressed as the effect of allometry should be minimal for samples from specific organs (Montgomery and Mank 2016), and the results are cross‐checked with top SNPs, making the analysis conservative. The associated scripts and final gene set are available at https://github.com/parisveltsos/feminisation_direction and http://www.doi.org/10.17605/OSF.IO/Z7FM9.

We used this list to ask if top SNPs colocalized with genes that are differentially expressed between the lines and if these also show different levels of diversity (Tajima's *D*) or differentiation (*F*_ST_) between E and M lines. We used a resampling approach, sampling genes (without replacement) from the *D. pseudoobscura* annotation, to determine the amount of overlap with the DE genes that is expected by chance. For each sample, we picked a set of 428 genes from the annotation, which is the same size as the set of genes near top SNPs (see Results). We then calculated the proportion of these genes that also occur in the DE gene sets and repeated this procedure 1000 times to build a distribution of expected overlap between resampled gene sets and the DE gene sets. If the empirical set of genes near top SNPs had a proportional overlap ≥ the 95^th^ percentile of the resampled distribution, it was deemed a “significant” overlap.

Using the values of Tajima's *D* and *F*_ST_ computed for each gene (see above), we also asked whether there was any evidence of different levels of diversity or divergence between DE genes in any set (*N* = 3173) and non‐DE genes (*N* = 13,583). For Tajima's *D*, we contrast DE and non‐DE genes separately for each chromosome type (autosomes, X‐chromosome left arm, and X‐chromosome right arm), and each experimental evolution treatment (E and M; six contrasts in total), using Wilcoxon rank sum tests. For *F*_ST_, we contrast DE genes and non‐DE genes separately for each chromosome type (three contrasts), testing for differences with Wilcoxon rank sum tests. In both cases, the mean value for non‐DE genes was used as a single value against which to compare DE genes, which reduces the effect of the enormous sample size for the non‐DE genes on the significance of the test.

Finally, we also asked whether the changes in sex‐biased expression (data from Veltsos et al. 2017) between E and M treatments (ΔSB_EM_) were related to diversity (Tajima's *D*) within either E or M lines. Sex bias in expression was assessed for two tissues, head and abdomen, in both courted or virgin data combined. Within each tissue, sex bias was computed as the log_2_(fold change) in expression between males and females in E and M lines separately, after which ΔSB_EM_ is calculated as log_2_(FC)_E_ – log_2_(FC)_M_. Thus, positive values of ΔSB_EM_ correspond to greater male bias in expression in the E lines, whereas negative values correspond to greater male bias in the M lines. ΔSB_EM_ was then related to values of Tajima's *D* in either E (TajD_E_) or M (TajD_M_) lines. For each tissue (head and abdomen), we performed an ANCOVA with chromosome (autosome, X‐chromosome right arm, and X‐chromosome left arm) as a co‐factor, as well as mean Tajima's *D* across E lines and mean Tajima's *D* across M lines as co‐variates. We also included the interactions between Tajima's *D* and chromosome. The full model is as follows:

$$\begin{array}{ccc} \Delta SB_{EM} & ∼ & chromosome+TajD_{E}+TajD_{M}\\ & & +TajD_{E}:chromosome+TajD_{M}:chromosome. \end{array}$$

We further extracted the 30‐bp up‐ and downstream of each SNP from the reference genome using gffread from the Cufflinks package (version 2.2.1; Trapnell et al. 2010) and tested for an enrichment of TF binding site motifs around top SNPs with the AME routine from the MEME package (version 4.10.2; McLeay and Bailey 2010). GO term enrichment analysis was performed with GOwinda (version 1.12; Kofler and Schlotterer 2012). We considered SNPs to be associated with genes if they occurred within 10‐kb up‐ or downstream of an annotated gene. An empirical *P*‐value distribution was produced from 1 million simulated SNP sets.

All statistical analyses were made with R (version 3.6.3; R Development Core Team 2020) except where otherwise stated. Figures were drawn using the “ggplot2” package (version 2.2.1; Wickham 2009) and associated packages (Table S1).

## Results

### CONSISTENT ALLELE FREQUENCY DIFFERENCES

**Figure 1:**
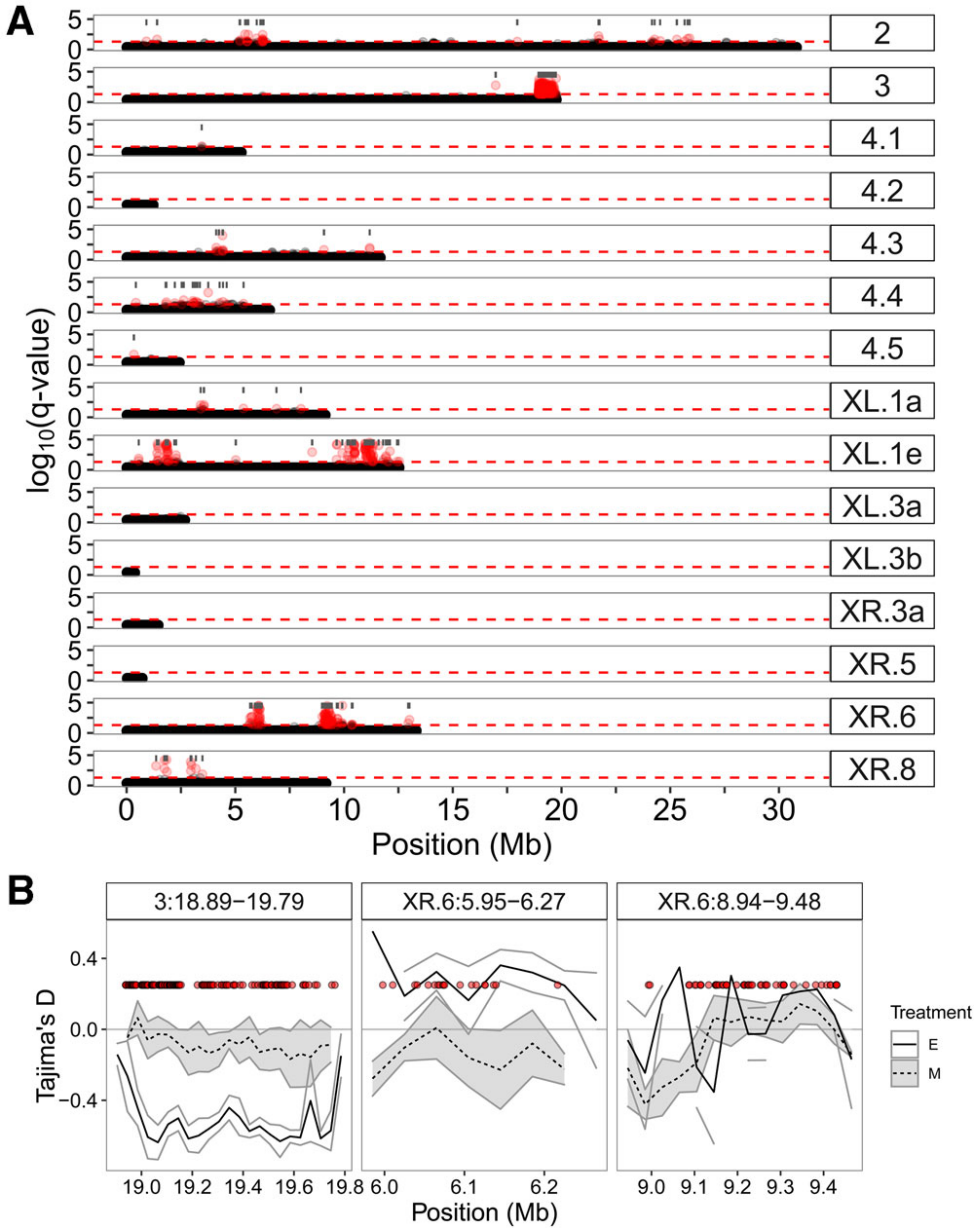
(A) Manhattan plot of log10(*q*‐values) for each SNP from a quasibinomial GLM with treatment as a predictor. Red points denote SNPs with a *q*‐value < 0.05 and the horizontal red dashed line indicates the *q* < 0.05 cutoff. Gray bars give the locations and span of the 70 divergent regions (see text). (B) Mean (± SE) Tajima's *D* across replicates for the three most divergent regions (see text); red points denote SNPs with a *q*‐value < 0.05; all have been plotted at the same value on the *y*‐axis for convenience. For higher resolution figures, see Figures S1–S5.

In total, 480 SNPs show significant consistent allele frequency differences due to the experimental evolution treatment (hereafter the “top SNPs”). These occur on all of the main chromosomes, but many show striking co‐occurrence into a few clusters of highly differentiated SNPs (Fig. 1A). The distribution of the top SNPs across the genome is not random, with a significant excess on the third chromosome and both arms of the X chromosome (Table S3). In particular, a large cluster of differentiated SNPs are observed at the end of the right arm of chromosome 3 (Fig. 1A). Other large clusters occur on both arms of the X chromosome (Fig. 1A). If all top SNPs within 50 kb of others are grouped into clusters, this produces 70 distinct clusters throughout the genome (Fig. 1A). The majority of SNPs (72.9%) occur in only six clusters with >10 SNPs. Hereafter, we refer to these clusters as “peaks.”

Such clustered divergence is often seen in comparisons between natural species (Ravinet et al. 2017) but rarely in experimental evolution (e.g., Kauranen et al. 2019). We considered all 68 possible unique permutations of the treatment labels among SNP sets and always observed fewer SNPs with *P*‐values < 0.05 than in the original dataset (Fig. S6A). We are therefore confident that our approach reliably identifies SNPs with consistently different allele frequencies between the treatments. We also tested if the divergence was more clustered than random samples between the lines using the top SNPs from the above permutated data. We show that permuted datasets, where the correlation structure of allele frequencies at nearby SNPs is preserved, result in a similar number of clusters as the original data (Fig. S6B; for full details of the randomization tests, see the Supporting Information). In a smaller dataset, where we tested the effect of removing this correlation structure by permuting treatment labels for each SNP, rather than for all SNPs. The number of clusters is larger when the correlation structure is removed. Thus, clustering of SNPs occurs, in large part, due to this correlation in allele frequencies at nearby SNPs. We also examined if variation in coverage might be associated with calling clustered divergence. We compared coverage within these peak regions to 100 random genomic regions with a similar distribution in size and found that, although there is a minor difference in coverage between peaks with top SNPs, the variation in coverage across samples is far greater. We therefore conclude that difference in coverage around top SNPs and the rest of the genome cannot explain the patterns (Fig. S7).

The peak regions do not correspond to known inversions in *D. pseudoobscura*. In particular, the large cluster on chromosome 3 containing many (*N* = 199, 41.5%) top SNPs does not correspond to the most common inversions that have shaped the evolution of this chromosome in the wild (Wallace et al. 2011, 2013). Allele frequencies in E and M lines for the top 100 SNPs are shown in Figure S8. More than half of these (57%) are fixed differences in all replicates. Across all the top 480 SNPs, 12% are fixed differences between the E and M lines in all replicates, with all of these occurring on the X chromosomes

### GENETIC DIVERSITY

We identified a set of candidate SNPs that vary consistently in allele frequency in response to experimental treatment. Such patterns are strongly suggestive of the action of selection. We therefore also assessed the levels of genetic diversity throughout the genome and in regions surrounding these candidates. On a broad scale, Tajima's *D* does not vary much across chromosomes (Fig. S9). Although Tajima's *D* is lower on chromosome 3 in E lines, there is no statistically significant overall effect of chromosome (*F*_4,30_ = 0.46, *P* = 0.76), and the interaction effect of chromosome and treatment is also not statistically significant (*F*_4,30_ = 0.59, *P* = 0.68). Strongly localized selective sweeps should locally reduce Tajima's *D*. Within E lines, Tajima's *D* is actually on average slightly higher within the clusters containing top SNPs (mean = −0.03) than outside these clusters (−0.05; Wilcoxon signed rank test: *V* = 17,623, *P*‐value = 0.04). Within M lines, there is no statistically significant difference between clusters (−0.07) and outside clusters (−0.06; *V* = 13,390, *P*‐value = 0.3). However, the broad similarity at a chromosomal level belies that patterns of Tajima's *D* are very variable between different chromosomal regions. The most differentiated region on chromosome 3 shows reduced Tajima's *D* within the E treatment compared to the M treatment (Fig. 1B), as would be expected following selective sweeps. Similar patterns are seen for some peaks on the X chromosome (Fig. S10). In a few cases, there are reductions of Tajima's *D* associated with peak regions containing top SNPs within M lines compared to E lines (Figs. 1B and S10). However, many of these regions are quite small and consequently estimates of Tajima's *D* may be unreliable (Fig. S10).

**Figure 2:**
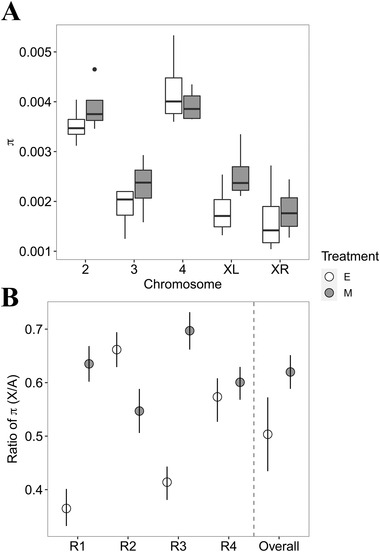
(A) Levels of genetic diversity (π) on each chromosome in E and M lines. π is estimated in overlapping windows of 50 kb, then averaged across the chromosomes. Boxplots show the distribution of π on each chromosome across replicate experimental evolution lines. (B) The X chromosome to autosome ratio of π in the replicates of E and M lines and overall.

Nucleotide diversity across the chromosomes was estimated as π (Fig. S11). Diversity is lower overall in E lines than in M lines (Fig. 2A). Diversity varies significantly across chromosomes in both E and M lines (Fig. 2A; *F*_4,30_ = 29.3, *P* < 0.001), but the interaction with treatment is not significant (*F*_4,30_ = 0.98, *P* = 0.44). Lowest diversity (in both treatments) is seen on the more differentiated chromosomes (X and 3; Fig. 2A). Median π is marginally nonsignificantly lower within the clusters of M (*V* = 12,471, *P* = 0.05), but not E (*V* = 13,843, *P* = 0.19), lines. The ratio of diversity between the sex chromosome and autosomes is lower in E lines than in M lines, although this is variable across replicates (Fig. 2B). Overall, it seems like there is greater evidence for selective sweeps in E lines, especially for the X.

#### Genetic differentiation

**Figure 3:**
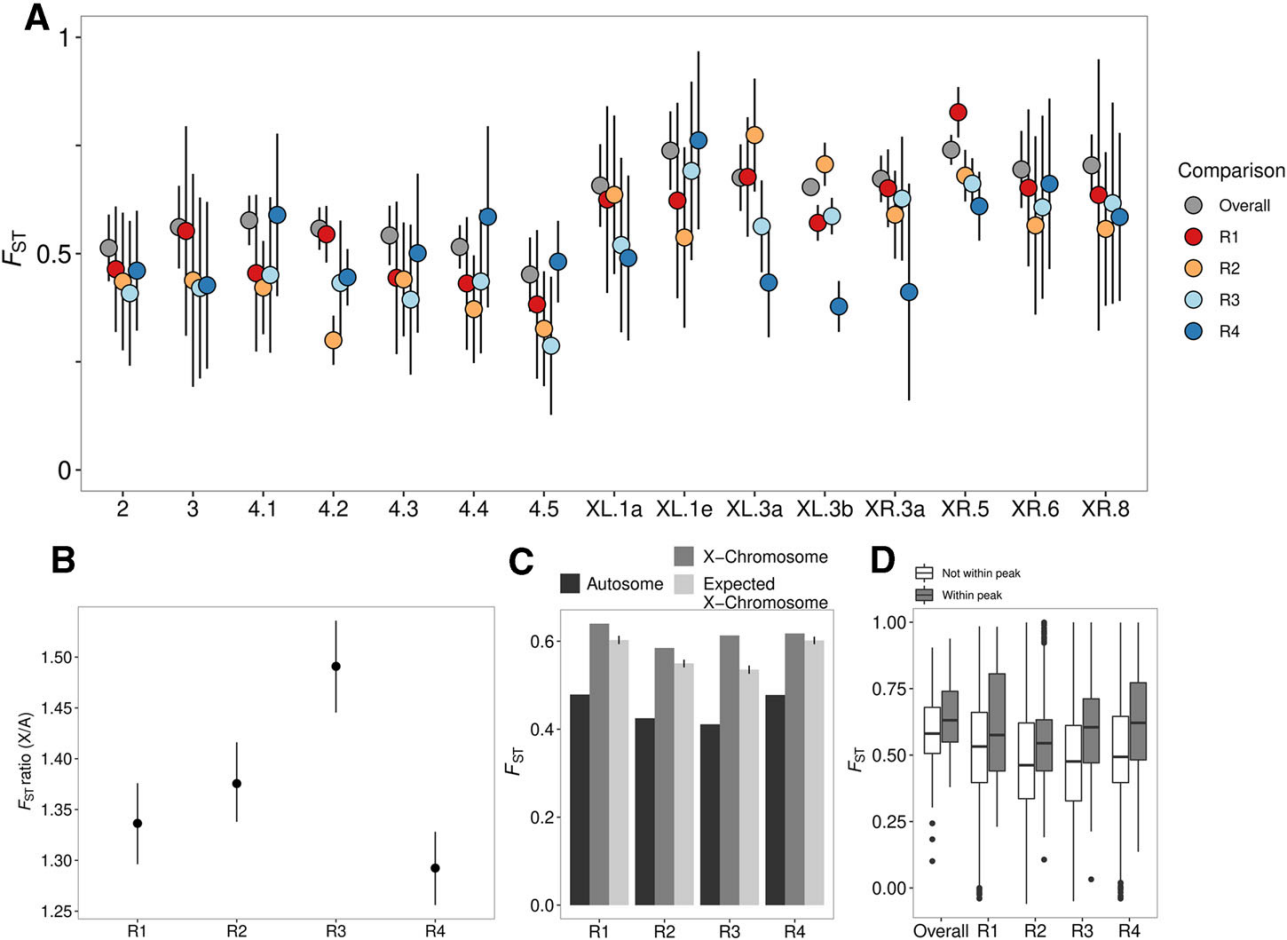
(A) *F*_ST_ between E and M treatment lines on the main chromosome arms for each replicate. *F*_ST_ is calculated for each SNP then averaged within overlapping 50‐kb windows on each chromosomal segment. (B) The X:autosome ratio of *F*_ST_ within each replicate line. The error bars are bootstrap 95% confidence intervals. (C) Observed *F*_ST_ on the autosomes (black) and on the X chromosome (dark gray) as well as the expected *F*_ST_ on the X chromosomes assuming a value of *z* = 6 (light gray) (see Methods); error bars represent bootstrap 95% confidence intervals. (D) The difference in *F*_ST_ between windows within “peaks” of top SNPs and windows outside of these peaks.

Comparisons of genomic divergence are often based on patterns of *F*_ST_. Although obviously not independent of changes in allele frequency, we also examined the patterns of *F*_ST_ seen between the E and M lines for comparison with published studies and to examine the X / autosome divergence in more detail. *F*_ST_ is generally higher on the X chromosome than on autosomes (Fig. 3B), even after accounting for the expected greater effects of drift on the X over the autosomes (see Methods for the equations; Fig. 3B). Hence, the X:A ratio of *F*_ST_ is always >1 (Fig. 3C). These results hold regardless of the ratio of breeding males to females (see Methods for the equations). *F*_ST_ was higher within peak regions than outside peak regions (0.64 vs. 0.59; Wilcoxon signed rank test: *V* = 15,309, *P*‐value < 0.001; Fig. 3D), as expected because allele frequencies differ most within the clusters. It should be noted that the above measures of differentiation and genetic diversity are often variable and precise estimates depend on the number of SNPs detected, the coverage, and number of replicate lines. Accordingly, we emphasize that although broad‐scale patterns are likely to be robust, values for any one genomic region or gene should be taken with caution.

### LINKAGE DISEQUILIBRIUM

**Figure 4:**
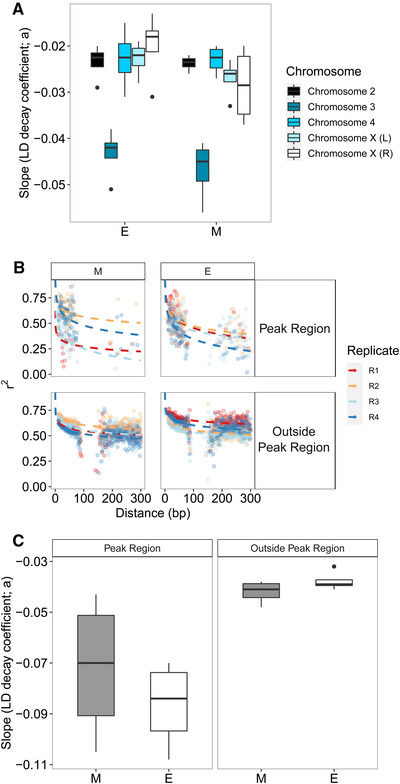
(A) Slope coefficients from the model *r^2^* ∼ *a* + log(*bp*), where *bp* is the distance between pairs of SNPs and *r^2^* is the average measure of LD between SNPs. Distributions are shown for average values of each of the main chromosomes as well as X chromosomes across replicates in E and M lines. (B) Decay in LD as a function of distance between SNPs with the chromosome 3 peak region (see Fig. 3) and outside the peak region for E and M lines. (C) The distribution of slope parameters for SNPs within the chromosome 3 peak and outside the peak region.

Background selection or selective sweeps could lead to clustered genomic divergence, often with low diversity, especially in regions of low recombination such as telomeric regions. We examined patterns of linkage disequilibrium in the clusters and if this varied with treatment. Throughout the genome, the decay rate (*a* parameter) of LD is generally shallower (i.e., less negative) in the E treatment (Fig. 4A). This is seen for chromosome 3 as well as both arms of the X chromosome (Fig. 4A). A lower decay rate is indicative of more LD, due to less recombination and/or a potential for greater hitchhiking under positive selection. Contrary to predictions, we found a steeper rate of decay (less LD) within the differentiated region of chromosome 3 than outside it, especially in E lines (Figs. 4B and 4C). Although statistically significant (*F*_2,13_ = 4.6, *P* < 0.001), these differences are slight and with much more variation across replicates in regions outside the peak. The most striking pattern overall is greater overall decline in LD on chromosome 3 (i.e., smallest value for the *a* parameter; *F*_4,34_ = 24.0, *P* < 0.001).

### GENE FUNCTIONS AND EXPRESSION VARIATION

Out of the 480 top SNPs, 201 (42%) lie within a gene model (i.e., either in an intron or within an exon); the remaining are intergenic. The top SNPs are not significantly enriched in any GO term after correcting for multiple testing, even at a 10% FDR (Table S4). Similarly, there is no enrichment of genes with annotations for mating behavior or stress response phenotypic classes. However, several genes within 10 kb of a top SNP are potentially interesting candidate genes for traits evolving under sexual selection based on described functions (Table S4). For example, the genes *Odorant‐binding protein 47a* (*Obp47*), *pickpocket 6* (*ppk6*), and *Accessory gland protein 53C14c* (*Acp53C14c*) all occur within 10 kb of a top SNP and are genes potentially underlying sexually selected behaviors or traits. Two of these genes (*ACP53C14c* and *Obp47a*) are within the region of highly differentiated SNPs on the third chromosomes, which also includes several additional accessory gland proteins (*Acp53Ea*, *Acp53C14b*, and *Acp53C14a*) and other genes (Table S4), all of which are thought to influence mating and courtship behaviors or phenotypes based on known functions of similar genes in *D. melanogaster*.

**Figure 5:**
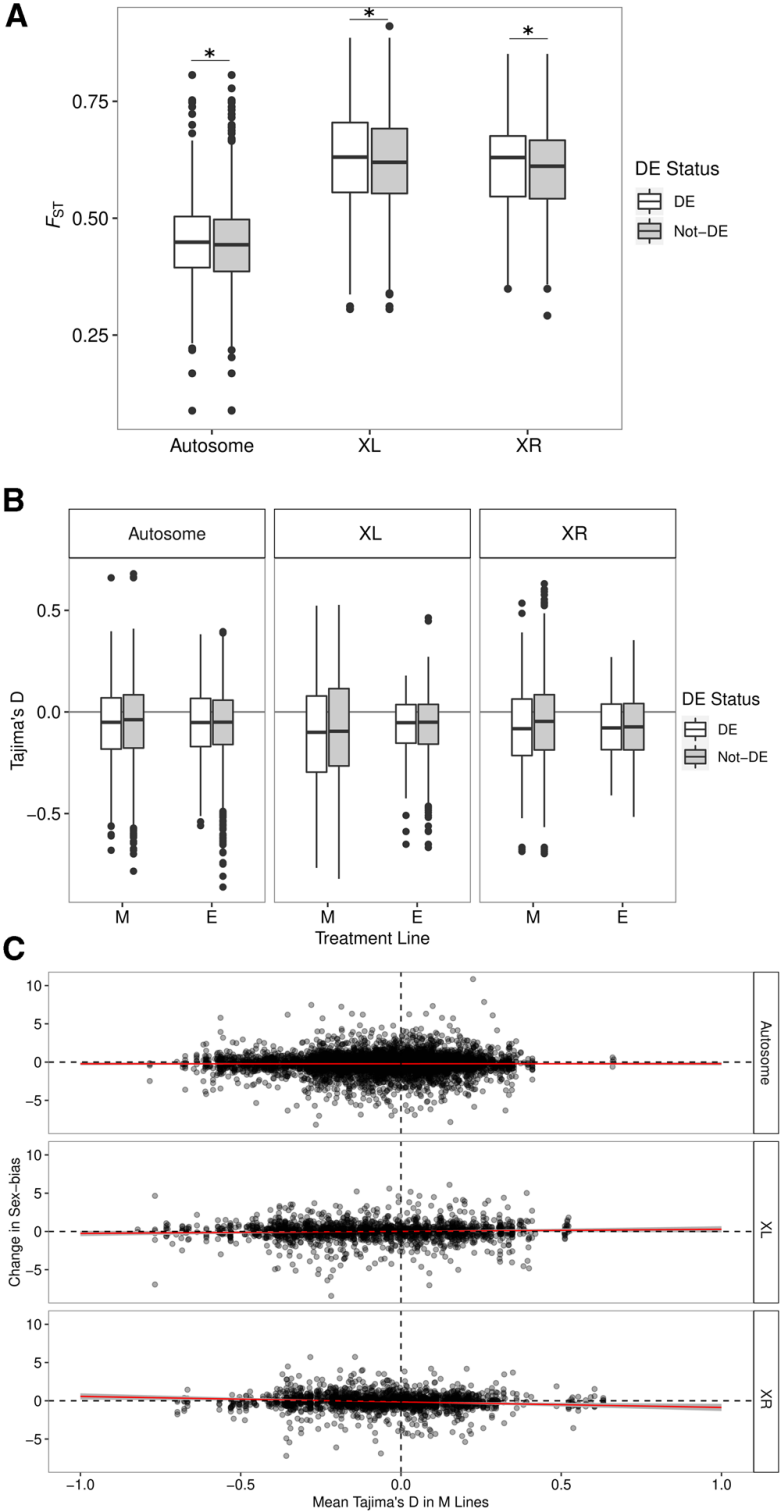
(A) *F*_ST_ at DE versus non‐DE genes for different chromosome types. Asterisks indicate significant differences. (B) Tajima's *D* at DE versus non‐DE genes for different chromosome types. (C) Relationship between change in sex‐bias between E and M lines and Tajima's *D* in M lines.

Previous studies have shown that there is divergence in gene expression patterns between E and M lines (Immonen et al. 2014; Veltsos et al. 2017, 2021.). We therefore asked if these expression differences were associated with the top SNPs. Genes within 10 kb (*N* = 428) of the top SNPs show a significantly greater overlap with genes that are differentially expressed (DE) in ovaries and testes between E and M lines than expected by chance (Fig. S12 and Table S3). This pattern also holds for genes within 1 Mb (*N* = 7045; Fig. S13). Also, there is evidence that *F*_ST_ between E and M lines is higher for genes that are DE between the lines, especially for X‐linked genes (Fig. 5A; Wilcoxon rank sum tests, Autosomes: *V* = 1,026,000, *P* = 0.03; X‐chromosome right arm: *V* = 89,067, *P* = 0.005; X‐chromosome left arm: *V* = 59,623, *P* = 0.04). There is no evidence that Tajima's *D* is different between DE and non‐DE genes (Wilcoxon rank sum test, all *P* > 0.05; Fig. 5B). There is some evidence that the degree to which sex‐biased expression of a gene changes between E and M lines is associated with Tajima's *D* in M lines, but only on the X‐chromosome and only within abdominal tissues (Fig. 5C). Specifically, as the change in sex bias becomes more negative (i.e., more female‐biased expression in M lines), Tajima's *D* also becomes more negative (interaction of Tajima's *D* in M lines and chromosome type: *F*_(11,189, 11,191)_ = 4.4, *P* = 0.01).

The regions immediately up‐ or downstream of top SNPs are not enriched for TF binding motifs or lncRNAs, after correction for multiple testing, so there were no obvious differences between treatments in regions expected to influence gene expression variation.

## Discussion

There is much debate about the influence of sexual selection and sexually antagonistic selection on patterns of genomic variation (Mank 2017; Sayadi et al. 2019) and how this may influence divergence between species (Wolf and Ellegren 2016). Sex‐biased gene expression, especially male bias, evolves quickly and is related to phenotypic sexual dimorphism (Wright et al. 2019). Outliers in genome scans often implicate sexual selection as a diversifying force (Andres et al. 2008; Blankers et al. 2018). Some signatures of sexual antagonism may be associated with genomic signatures of selective sweeps or balancing selection (Cheng and Kirkpatrick 2016; Wright et al. 2019) and may be promoted by strong sexual selection (Connallon and Clark 2012, 2013; Dutoit et al. 2018; Ruzicka et al. 2019). However, inferences of the sources of selection on natural variation in genomic divergence are usually indirect and ambiguous, because multiple forces act in concert to produce variation seen at the genomic level in nature. Here, we used experimental evolution to alter sexual selection intensity, elevating sexual selection in polyandrous lines and eliminating it in monogamous lines, and examined patterns of divergence in the genome after more than 160 generations of experimental evolution.

Many of the results we found recapitulate patterns seen in natural populations and between species. Divergence is not uniform across the genome but clustered in “islands” of divergence, some of which contain candidate genes for an involvement in mating success. These clusters are more often seen on the X chromosome, which is a “hotspot” for divergence. There are signatures of selection within the islands of divergence, with marginally lower diversity (π) within clusters than the rest of the genome, but only in M lines. *F*_ST_ between E and M lines is greater within clusters, and is also greater on the X than autosomes, and differences in diversity are seen in the autosomes between selection regimes. Low Tajima's *D* implies selective sweeps have occurred, but only within some of the divergent regions. These patterns of diversity and divergence are associated with changes in both differential gene expression between the lines and sex‐biased genes. Overall, *F*_ST_ between the lines is high in all replicates, probably due to low overall effective population sizes, although effective population sizes are similar between E and M lines (Snook et al. 2009).

The concept of “islands” of divergence originated from comparisons of genomic divergence between species (Nosil et al. 2009; Ravinet et al. 2017). These are usually thought to have arisen due to the combination of strong selection on barrier loci and genetic hitchhiking within genomic regions, with background gene flow reducing divergence outside of the islands. Here, we find distinct clustered divergence akin to the islands seen in natural systems. Our system is effectively allopatric, so there was no background gene flow counteracting divergence outside of these clusters, which therefore must have arisen due to strong localized divergent selection across all replicates. Although *D. pseudoobscura* has relatively well‐characterized inversion polymorphisms (Sturtevant and Dobzhansky 1936; Dobzhansky and Sturtevant 1938; Wallace et al. 2011), the clusters we describe do not correspond to the most common inversions known for this species, which are often very large. Our short‐read sequencing approach allowed some examination of LD and there was no suggestion of reduced recombination within the clusters. In fact, the large peak at the right end of chromosome 3 (Fig. 4) surprisingly seems to be within a region of high recombination (which is often suppressed at telomeric regions). Interestingly, recombination is higher within this peak than the chromosome‐wide rate, but also differs between the treatments, being greater in the M lines. Perhaps selection against recombination was reduced in monogamous individuals because of epistatic interactions in the region that were important in sexual selection or sexual conflict. There was no obvious difference in LD in the other clusters but their smaller size and hence “noisier” estimates make robust inferences from pool‐seq data difficult. Indeed, the estimates of LD within the cluster on chromosome 3 also rely on relatively few SNPs at longer ranges compared to the rest of the chromosomes, so inferences need to be taken with caution.

The lack of background gene flow or stronger linkage disequilibrium within the clusters suggests that they have arisen primarily through localized strong selection that is consistent across all replicates, although localized hitchhiking is likely to be primarily responsible for the clusters. In support of this, we see lower Tajima's *D* in some of the larger clusters. However, these patterns are very variable with lower Tajima's *D* in different clusters for the E and M lines. Thus, overall, there is no significant difference in Tajima's *D* between E and M lines. Systematic differences in Ne between E and M lines might be expected to lead to consistent differences in Tajima's *D*. One might predict lower *N*_e_ in M lines due to fewer mating individuals and, correspondingly, lower Tajima's *D* in M lines, although the experimental design tried to minimize this and previous studies found no evidence of such a reduction in *N*_e_ (Snook et al. 2009).

The genes contained within the clusters are not enriched in particular functional categories; however, they include strong candidate genes for an involvement in mating system evolution. For example, the large region on chromosome 3 contains numerous accessory gland proteins. In *D. melanogaster*, these are well known to influence male reproductive success, exert antagonistic effects on female fecundity and lifespan, and play a role in sperm competitive success (Chapman et al. 1995; Ram and Wolfner 2007). Some of the evolutionary response in E lines is antagonistic, because M females have a lower fecundity when mated with E males. Moreover, when mated to E males, the reproductive schedule of M females is manipulated to the males benefit (Crudgington et al. 2010). Accessory gland proteins show accelerated coding sequence and gene expression evolution across species (Swanson and Vacquier 2002; Begun and Lindfors 2005). Other genes within the clusters are involved in sexual chemical communication, which is also often implicated in outlier analyses in genome comparisons between species (Smadja and Butlin 2009). For example, mutants of members of the pickpocket family in *D. melanogaster* show aberrant male mating success because of their involvement in the detection of female pheromones (Thistle et al. 2012; Toda et al. 2012). E males, subject to both intra‐ and intersexual selection, have diverged in aspects of courtship behavior, such as time until initiation of courtship, have a higher intensity courtship song, and have a higher competitive mating success than M males (Debelle et al. 2016; Debelle et al. 2017).

If strong selection has driven this clustered genomic divergence, an interesting question is whether the responses to selection are stronger in the E or M lines. Imposing monogamy on a naturally polyandrous species probably leads to relaxed selection on many genes involved in intra‐ or intersexual competition. Therefore, the response is likely to involve changes in both the intensity and direction of selection on some loci. Thus, perhaps the variation in signals of selection we see in Tajima's *D* and changes in LD are to be expected. Overall, we see stronger reductions in diversity in E lines, perhaps suggesting that directional selection was stronger when sexual selection was strengthened.

One pattern very commonly seen in studies of natural populations and species is more rapid divergence of the X chromosome (Vicoso and Charlesworth 2006). We also see this here, the X having a higher prevalence of divergent clustered regions and consequently higher *F*_ST_ between the lines. Remarkably, all SNPs with fixed differences between the lines occurred on the X. Faster X evolution can occur for many reasons, including greater genetic drift due to its smaller effective population size, and beneficial recessive alleles on the X are more responsive to selection due to male hemizygosity (Meisel and Connallon 2013). We calculated expected X/A divergence ratios under a range of plausible sex ratios and the observed X/A divergence exceeded all of them, suggesting the accelerated X divergence is not due to drift effects alone, selection or a combination of effects is likely involved. Genes under sexual selection are potentially more likely to be sex linked, due to antagonistic, or sex‐limited selection (Reinhold 1998; Kirkpatrick and Hall 2004). Sexually selected or antagonistic loci are perhaps also more likely to show dominance reversal effects (Grieshop and Arnqvist 2018).

Previously we found that gene expression differences have evolved between the lines, especially in sex‐biased genes (Veltsos et al. 2017). Here, we show that there is significant overlap between differentially expressed genes and the regions of genomic divergence of the lines found here. Thus, the expression divergence is associated with the broad patterns of genomic divergence. Also, *F*_ST_ is greater for the differentially expressed genes, once again recapitulating patterns from natural systems (sex‐biased genes here are not more likely to be sex linked, so this is independent of the large X effect seen). We find no overall difference in Tajima's *D* between DE and non‐DE loci.

Links between differences in gene frequencies between the sexes, genomic parameters, and sex‐biased gene expression variation have been a somewhat contentious source of evidence of sexual selection, especially antagonistic forms of sexual selection (Kasimatis et al. 2019; Cheng and Kirkpatrick 2020; Mank et al. 2020). Genes that are male biased in expression show accelerated divergence between species and sex‐biased gene expression shows rapid evolution and turnover (Pröschel et al. 2006; Harrison et al. 2015). Whether sex‐biased expression is expected to be related to sex‐specific *F*_ST_ or signatures of balancing selection such as Tajima's *D* is open to debate, partly because of the potential resolution of antagonistic selection by the strengthening of sex‐biased expression. However, there is one very intriguing pattern in our data where the magnitude of change in sex‐biased gene expression is related to Tajima's *D*. As ΔSB increases (e.g., more male‐biased expression in E lines), Tajima's *D* in these lines becomes more negative. This pattern is potentially consistent with more resolved sexual conflict in the M lines, because males in M lines are released from sexual selection, and selection driving female‐beneficial alleles to high frequency could result in sweeps and/or reduced balancing selection. However, perhaps analyses over the course of the experimental evolution study would be required to convincingly demonstrate associations between changes in sex bias and potential measures of balancing selection.

In conclusion, we have examined genomic divergence following >160 generations of experimental evolution under altered mating systems. We find that genomic divergence between the experimental lines is highly clustered in the genome, much greater on the X, and is associated with changes in gene expression between the experimental lines. Associations with LD and population genetic parameters indicative of selective sweeps or balancing selection are also observed, but are very variable. This raises the possibility that selection has been strong in both M and E lines, but differs in nature (relaxed in M, directional in E), complicating predictions of responses. Overall, our main results support those seen in natural populations, providing an elegant demonstration of the power of experimental evolution to aid the interpretation of complex patterns of natural variation.

## AUTHOR CONTRIBUTIONS

RAWW performed the data analysis. PV contributed data. The experiment was designed by MGR and RRS. All authors contributed to writing the manuscript.

## DATA ARCHIVING

Raw reads have been deposited in the short read archive (SRA) of NCBI under the BioProject PRJNA661678.

## Supporting information


**Table S1**. List of R packages and references.
**Table S2**. Coverage and mapping statistics.
**Table S3**. Distribution of chromosome lengths, the overall number of SNPs and the number of outlier SNPs across the main chromosome arms.
**Figure S1**. Manhattan plot of log10(q‐values) for each SNP from a quasibinomial GLM with treatment as a predictor on chromosome 2.
**Figure S2**. Manhattan plot of log10(q‐values) for each SNP from a quasibinomial GLM with treatment as a predictor on chromosome 3.
**Figure S3**. Manhattan plot of log10(q‐values) for each SNP from a quasibinomial GLM with treatment as a predictor on the separate regions of chromosome 4.
**Figure S4**. Manhattan plot of log10(q‐values) for each SNP from a quasibinomial GLM with treatment as a predictor on the separate regions of the right arm of the X‐chromosome.
**Figure S5**. Manhattan plot of log10(q‐values) for each SNP from a quasibinomial GLM with treatment as a predictor on the separate regions of the left arm of the X‐chromosome.
**Figure S6**. A The distribution of the proportion of SNPs with a “treatment” effect that achieves a p‐value < 0.05 across 68 permuted SNP datasets.
**Figure S7**. Coverage distributions between the regions around top SNPs (peak regions) and 100 randomly sampled regions with a similar length distribution (random regions).
**Figure S8**. The allele frequencies in E and M lines for the top 100 SNPs with the lowest q‐values from a quasibinomial GLM of allele frequency differences.
**Figure S9**. Levels of genetic diversity (Tajima's *D*) on each chromosome in E and M lines.
**Figure S10**. Mean (±SE) of Tajima's *D* in overlapping 50 kb windows along the chromosomal regions underneath the 70 peaks of highly differentiated SNPs.
**Figure S11**. π in overlapping 50kb windows across chromosomes and replicates of E and M lines.
**Figure S12**. Distributions of overlap between bootstrap samples of genes and sets of differentially expressed genes from Veltsos *et al*., (2017 and 2021).
**Figure S13**. Distributions of overlap between bootstrap samples of genes and sets of differentially expressed genes from Veltsos et al., (2017 and 2021).

Supplementary Material
